# Generation of orientation tools for automated zebrafish screening assays using desktop 3D printing

**DOI:** 10.1186/1472-6750-14-36

**Published:** 2014-05-01

**Authors:** Jonas N Wittbrodt, Urban Liebel, Jochen Gehrig

**Affiliations:** 1 Acquifer AG, Karlsruhe, Germany; 2 Accelerator Laboratory, Innovation Department, Karlsruhe Institute of Technology, Eggenstein-Leopoldshafen, Germany

**Keywords:** Orientation tool, 3D printing, Zebrafish screening, High content screening

## Abstract

**Background:**

The zebrafish has been established as the main vertebrate model system for whole organism screening applications. However, the lack of consistent positioning of zebrafish embryos within wells of microtiter plates remains an obstacle for the comparative analysis of images acquired in automated screening assays. While technical solutions to the orientation problem exist, dissemination is often hindered by the lack of simple and inexpensive ways of distributing and duplicating tools.

**Results:**

Here, we provide a cost effective method for the production of 96-well plate compatible zebrafish orientation tools using a desktop 3D printer. The printed tools enable the positioning and orientation of zebrafish embryos within cavities formed in agarose. Their applicability is demonstrated by acquiring lateral and dorsal views of zebrafish embryos arrayed within microtiter plates using an automated screening microscope. This enables the consistent visualization of morphological phenotypes and reporter gene expression patterns.

**Conclusions:**

The designs are refined versions of previously demonstrated devices with added functionality and strongly reduced production costs. All corresponding 3D models are freely available and digital design can be easily shared electronically. In combination with the increasingly widespread usage of 3D printers, this provides access to the developed tools to a wide range of zebrafish users. Finally, the design files can serve as templates for other additive and subtractive fabrication methods.

## Background

Automated microscopy of zebrafish embryos and larvae has been established as a powerful methodology to study biological processes within the complexity of a vertebrate embryo at a larger scale. Applications range from lower resolution screening in toxicological or behavioral studies [1] to automated imaging at single cell resolution [2]. Regardless of the imaging modality, scoring of detailed morphological or cellular phenotypes is often complicated by inconsistent orientation of specimen, mainly caused by their complex three-dimensional shape. However, consistent and reproducible positioning is a prerequisite for the quantitative and comparative analysis of acquired image data in most assays.

To address this requirement, researchers have developed various protocols and tools that facilitate mounting and positioning of zebrafish embryos and larvae [3-9]. However, most solutions are incompatible with microtiter plates, which are the commonly employed sample holder for automated imaging using commercially available microscopes. To overcome this limitation, we have previously demonstrated orientation tools allowing the consistent acquisition of lateral and dorsal views of embryos arrayed within wells of microtiter plates [10,11]. However, a drawback of these solutions is that their distribution across laboratories can be difficult due to the relatively high cost of replication, or the lack of access to workshops with required machining capacities.

Recently, affordable additive manufacturing devices such as desktop 3D printers have emerged as part of a larger open source soft- and hardware community. In comparison to most standard laboratory equipment or industry-grade 3D printers, these devices are considerably cost-effective and can substitute for a large range of machining requirements in producing scientific hardware [12,13]. Moreover, novel models can be readily generated using open-source CAD software allowing rapid prototyping cycles, and digital design files can be easily shared or deposited in public open hardware databases [14].

Here, we demonstrate the utilization of desktop 3D printers to fabricate 96-well plate compatible orientation tools for zebrafish embryos enabling the acquisition of consistent lateral or dorsal views in screening assays using automated microscopy. The conceptual design of tools is based upon previously published work [10,11]. However, we have added novel features that improve overall functionality, handling and embryo positioning. Importantly, the utilized fabrication method is readily reproducible and digital 3D models can be easily shared, thus greatly facilitating access to the developed tools.

## Results and discussion

### Design of zebrafish orientation tools

Lateral and dorsal views of zebrafish embryos are routinely employed to evaluate and compare morphological phenotypes or reporter gene expression patterns in various assays. To this end, we focused on developing tools for these two standard orientations, which generate agarose molds within wells of microtiter plates that facilitate positioning and can stably hold oriented embryos and larvae.

**Figure 1:**
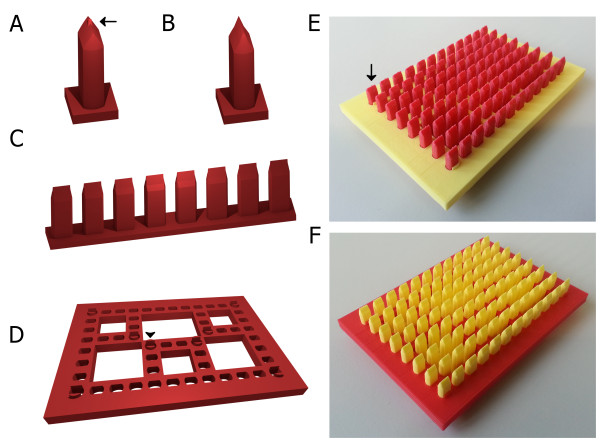
**Overview of digital design files for 3D printing of orientation tools.** Shown are images of renderings of the two pin designs for **(A)** lateral and **(B)** dorsal orientation, **(C)** a pin stripe and **(D)** the baseplate design. **(E, F)** Photographs of printed and assembled tool: **(E)** lateral and **(F)** dorsal. Arrows in **A** and **E** point to the small pin generating the mold for the yolk sac for lateral orientation. Arrowhead in **D** points to the clip used for anchoring the baseplate to the microtiter plate.

To ensure a flexible setup and save printing time, the tools were designed modularly consisting of a base plate and a set of stripes each harboring a row of pins (Figure 1A-D). To improve overall embryo positioning within wells of microtiter plates, the pins are designed to generate deep agarose cavities allowing a fixed anteroposterior orientation and reduce movement of embryo surrounding medium (Figure 1A-C). The basic shape of pins is a cylinder flattened on two sides, which tapers near the top of the pin. The pins end in geometries for generating different types of molds that support the lateral or dorsal orientation of embryos within cavities. These molds were designed by taking into account the size and shape of basic embryo features, such as the yolk and trunk, and were empirically optimized by iteratively modifying and testing different properties of the pins. The geometries resemble previously demonstrated designs: (i) a pin shaped geometry allowing a lateral positioning by holding the yolk ball (Figure 1A) [10], and (ii) a keel shape geometry for ventral positioning of zebrafish embryos and larvae (Figure 1B) [6,11]. The latter allows the acquisition of dorsal views on inverted microscopes.

The base plate contains slots for holding the pin stripes (Figure 1C, D). The shape of slots matches the contour of the pins of both designs, ensuring a stable x-y-fixation. The base plate also carries 8 clips to anchor the orientation tool at the microtiter plate and aid in accurately positioning the pins within wells. To create the final stamp tool, the pin stripes are slid into the base plate with a pin for each well of a standard 96 well microtiter plate (Figure 1E, F).

### 3D printing setup for zeb*r*afish orientation tools

To generate corresponding digital designs, 3D objects were modeled using the free software OpenSCAD [15] and processed using ReplicatorG [16]. The models were printed on a MakerBot Replicator 2 (MakerBot® Industries, USA) desktop grade 3D printer.

To optimize print quality and improve reproducibility of results, several modifications were made to the 3D printer: the extruder was upgraded to improve feeding of filament (thing:35810) and the original fan duct was replaced to optimize printing of fine detail (thing:51426). The required parts for both upgrades were simply printed using the original non-modified printer. These installments improved surface finish and accomplishable level of detail leading to a higher quality agarose molding and thus more reliable positioning of embryos. To improve flatness of large prints, the original build plate was replaced by a 4 mm aluminum plate elevated by a custom designed spacer (see Additional file 1). Flatness of both, the build plate and pin stripes, is crucial for screening applications as it minimizes variations in z-positioning of embryos within different wells of the microtiter plate, thus reducing required z-ranges for autofocusing or z-stack sizes.

Due to the nozzle diameter of 0.4 mm, the lateral print resolution of the MakerBot Replicator 2 is too coarse to produce the detail required for pin geometries matching the dimensions of zebrafish embryos and larvae. Therefore, the pin stripes were designed to print at an angle to utilize the better z-resolution of 100 μm. If reproduced on a 3D printer, or other fabrication method, with a lateral resolution better than 100 μm the angled production can potentially be omitted. With all these optimizations applied the Replicator 2 offered satisfactory level of detail and sufficient reproducibility of results (Figure 1E, F).

### Automated acquisition of dorsal and lateral views of zebrafish embryos

To verify the utility of the 3D printed orientation tools in zebrafish screening assays, we carried out imaging experiments to automatically acquire lateral and dorsal views of 48 hpf zebrafish embryos. Therefore, agarose coated microtiter plates were prepared according to reference [11]. In brief, 1% agarose was filled into 96-well microtiter plates and cavities were formed by inserting the assembled orientation tools. After solidification of the agarose, the tool was carefully removed. Embryos were anesthetized using tricaine and plated into wells. Zebrafish embryos were manually oriented under a stereomicroscope and imaged on an inverted screening microscope (see Additional file 2).

**Figure 2:**
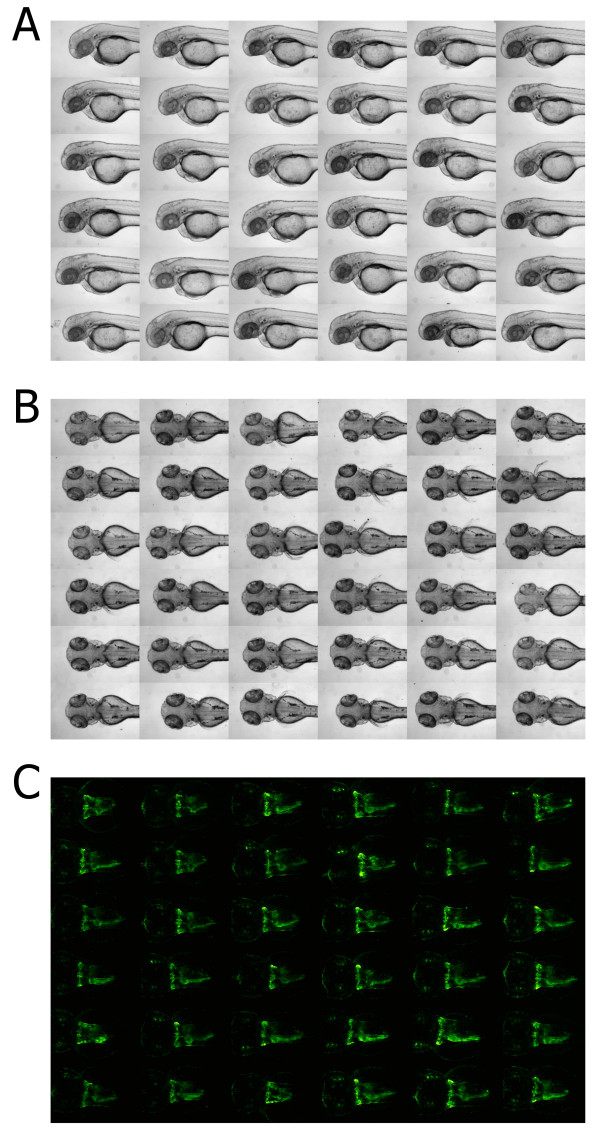
**Screening data obtained using the 3D printed orientation tools.** Shown are illustrative examples of embryos within agarose cavities generated with 3D printed orientation tools. All images shown derive from single 96 well plates with laterally or dorsally oriented embryos, respectively (see also Additional file 3). **(A, B)** Cropped extended focus bright field images of 48 hpf zebrafish embryos: **(A)** lateral and **(B)** dorsal views. **(C)** Cropped maximum projections of deconvolved z-stacks of kidney regions of 48 hpf embryos of the *Tg(wt1b:EGFP)* transgenic line.

As shown in Figure 2 the plates produced with the 3D printed tools can reliably hold specimen after positioning, and can be used to obtain consistent lateral (Figure 2A) and dorsal views (Figure 2B) of embryos. Besides geometry design, the percentage of embryos that obtain and preserve the desired orientation is also dependent on the manual skill of the experimenter; however in a routinely prepared plate the vast majority of embryos will be properly positioned (see Additional file 3). Additionally, the design of both tools enable to position specimen in a fixed anteroposterior orientation providing further standardization. For lateral orientation, this represents substantial improvement over a previously demonstrated protocol, where standardized anteroposterior orientation of embryos could only be achieved *in-silico* using a custom designed image processing pipeline [10]. Moreover, the deep keel shape cavity harboring the embryos in both designs reduces movement of surrounding medium. This greatly stabilizes overall embryo positioning, thereby enabling the usage of stacking robots and improving general sample and plate handling in zebrafish screening assays. To test the applicability in fluorescence imaging, we carried out automated imaging of dorsal views of larval kidneys of the *Tg(wt1b:EGFP)* stable transgenic line [17] (Figure 2C). Importantly, no background fluorescence could be observed caused by potential traces of printing material. To assess the applicability of the plates for continuous monitoring of specimen, we imaged *Tg(wt1b:EGFP)* embryos over a period of 29 hours. No apparent overall morphological or developmental defects, or malformations of the developing kidney could be observed (see Additional file 4). The tool for dorsal imaging presented here reproduces previously demonstrated results which were obtained using a similar tool generated by CNC milling [11]. Thus, despite a less accurate surface finish and level of detail, the obtained exactness of 3D printed tools is fully sufficient to prepare plates for acquiring reproducible screening data.

## Conclusions

Here, we have shown that desktop 3D printers can be used for the production of zebrafish orientation tools that facilitate the scoring and comparative analysis of morphological phenotypes or reporter gene expression patterns in automatically acquired datasets. The tools can replace other, previously demonstrated devices [10,11], with the added benefit of cost-efficient production and facilitated accessibility by sharing of digital models. Moreover, novel features have been implemented that aid in general sample positioning, plate preparation and handling. The current designs require manual positioning of specimen. While being significantly faster than alternative mounting methods, this can still cause a significant work load when a large number of plates needs to be processed. Nevertheless, we have demonstrated that similar plates can be efficiently used in medium to large scale screening experiments [10,11]. Although systems for fully automated orientation and imaging exist, they usually require a sophisticated technical setup [7]. In contrast, the plates demonstrated here allow to readily conduct complex assays on any microscope compatible with standard microtiter plates and can be easily handled by most laboratories. The compatibility to microtiter plates also allows the usage of common sample handling devices such as stacking robots, liquid handlers or embryo pipetting robots for automated transferring of embryos into wells of microtiter plates [18,19].

The presented tools have been designed for 48–72 hpf zebrafish. For other developmental stages, orientations or model systems (e.g. medaka), the pin geometries potentially need to be adjusted. However, the design files (see Additional file 5) can be readily modified using free software to target other applications. Moreover, being a cost-effective and readily accessible rapid prototyping technology, desktop 3D printing should enable other researchers to rapidly design and implement novel models. All OpenSCAD and corresponding STL design files presented here are freely accessible (see Additional files 1, 5) and can be easily shared electronically. As 3D printers are becoming increasingly widespread, especially at universities and companies, this provides access to the developed tools to a wide range of zebrafish users [9]. Finally, the presented designs can serve as templates for other additive or subtractive manufacturing techniques.

## Methods

### Modelling and 3D printing

3D objects were modelled using OpenSCAD [15]. Models were exported as STL-files and processed using ReplicatorG [16]. 3D printing was carried out on a MakerBot Replicator 2 (MakerBot® Industries, USA) using polylactic acid (PLA) filament. Raft and support structures were not used. The extruder was modified after thing:35810 and the original fan duct was replaced by thing:51426. The original build plate was replaced by a 4 mm aluminum plate elevated by a spacer (see Additional file 1). The build plate was covered with painters tape to ensure adhesion of prints. The pin stripes were printed at an angle of 30° with 100 μm layer height, 15% infill and four outer shells. The base plate was printed with 250 μm layer height, 10% infill and 2 outer shells. The base plate was printed with 54 holes to reduce printing time. Total print time was about 14 hours (12 hours for 12 pin stripes and 2 hours for the baseplate). If printed on a different 3D printer the pinstripes require at least 100 μm z-resolution and enough shells to be waterproof. On 3D printers with a minimum feature size better than 100 μm the pinstripes can potentially be printed upright.

### Preparation of agarose coated microtiter plates

The procedure for preparing agarose coated microtiter plates was carried out according to reference [11] with minor modifications (see Additional file 2). 50 μl of hot 1% agarose in fish water or embryo medium was added to each well of a 96 well microtiter plate using a multi-channel pipette and pre-cooled at room temperature for 1 minute. The 3D printed orientation tools were inserted and attached to the microtiter plate using the guidance clips present at the base plate. After solidification of the agarose the tool was carefully removed. Prior to transferring into wells of microtiter plates, PTU treated embryos were anesthetized using 0.03% tricaine. Embryos were manually transferred in a volume of 100–150 μl using a cut 200 μl tip, and arrayed and oriented under a stereomicroscope using a bend injection needle [11]. The entire procedure of manual embryo loading and arraying requires approximately 15–20 min per 96 well plate.

### Automated imaging and image processing

Imaging was carried out on a standard Scan^R high content screening microscope (Olympus, Hamburg, Germany) [20] as previously described [6,10,11]. To compensate for minor variations in z-positioning and ensure capturing of entire specimen, z-stacks were used for automatic acquisition. Brightfield images were acquired using 6 z-slices, dz = 55 μm and a 2.5× (N.A. = 0.08) objective. Fluorescence images of the kidney region of the *Tg(wt1b:EGFP)* embryos were acquired using 33 z-slices, dz = 15 μm and a 4× (N.A. = 0.13) objective. Timelapse experiments were carried out on an Acquifer IM02 (Acquifer, Karlsruhe, Germany) using 33 z-slices, dz = 15 μm and a 4× (N.A. = 0.13) objective. Images were automatically cropped using a Fiji [21] macro modified after reference [11]. Fluorescence z-stacks were batch deconvolved using Huygens Professional (SVI, Hilversum, The Netherlands).

### Availability of supporting data

All digital design files are provided as Additional information.

## Abbreviations

3D: Three dimensional; CNC: Computerized numerical control; HCS: High content screening; hpf: Hours post fertilization; PLA: Poly lactic acid.

## Competing interests

JNW, UL and JG are employed by Acquifer AG.

## Authors’ contributions

JNW generated CAD models and carried out 3D printing, JNW and JG designed the orientation tools, UL and JG conceived and designed the study, UL and JG supervised the study, JNW and JG wrote the manuscript. All authors read and approved the final manuscript.

## Supplementary Material

Additional file 1 STL files of orientation tool designs. Click here for file

Additional file 2 Protocol 1: Step-by-step protocol for generating agarose coated microtiter plates and orientation of embryos using 3D printed orientation tools. Click here for file

Additional file 3: Figure S1 Examples of data from entire 96 well plates containing oriented embryos. Shown are laterally and dorsally oriented embryos from two single 96 well plates. (A-D) lateral orientation and (E-H) dorsal orientation. To illustrate average orientation accuracy within a single plate originating from a routine experiment, embryos were divided in three categories: (A, E) well oriented (lateral: 92.7%, dorsal: 78.1%), (B, F) slightly tilted (lateral: 6.3%; dorsal: 16.7%) and (C, G) failed orientation (lateral: 1.0%; dorsal: 5.2%). Embryos damaged during pipetting or orienting were included in the failed category. Thumbnail images in D and H show overviews of entire 96 well plates. Click here for file

Additional file 4: Movie S1 Continuous monitoring of oriented embryos. Shown is a single dorsally oriented embryo from a 96 well plate acquired over a period of 29 hours. Click here for file

Additional file 5 OpenSCAD files of orientation tool designs. Click here for file
