# Supplementary material for: Generation of orientation tools for automated zebrafish screening assays using desktop 3D printing
Source: BMC Biotechnol. 2014 May 1;14:36. doi: 10.1186/1472-6750-14-36 (PMC4021294; doi:10.1186/1472-6750-14-36)
Supplement: Additional file 2 — Protocol 1: Step-by-step protocol for generating agarose coated microtiter plates and orientation of embryos using 3D printed orientation tools. [file 1472-6750-14-36-S2.pdf]

Step-by-step protocol for generating agarose coated microtiter plates and orientation of embryos using 3D printed orientation tools

Additional information for:

Generation of orientation tools for automated zebrafish screening assays using desktop 3D printing

Jonas N. Wittbrodt[1], Urban Liebel[1,2], Jochen Gehrig[1,2*]

[1]Acquifer AG, Karlsruhe, Germany;

[2]Accelerator Laboratory, Innovation Department, Karlsruhe Institute of Technology, Eggenstein-Leopoldshafen, Germany;

Email addresses: Jonas.Wittbrodt@posteo.de (J.N.W.), u.liebel@acquifer.de (U.L.), j.gehrig@acquifer.de (J.G.).

Procedure

3D printing of orientation tools

Using the OpenSCAD scripts

If you do not want to change any settings use the STL files as provided and proceed with step 5.

1. Download all three .scad files and put them into the same folder.
2. To create the baseplate STL file, open the baseplate.scad using OpenSCAD.
3. Render (F6) and export as STL (Design -> Export as STL…).
4. To generate the pin stripe STL files, open the pin.scad file.

    a. Optional: Adjust the 'prepare print' command according to the syntax guide included in the file.
    b. It is recommended to print four stripes at once, more are possible, but may require STL cleaning and/or a powerful machine to render.
    c. Render the pinstripes and Export as STL as described above.

Printing the orientation tool

5. Connect the Replicator 2 to the computer. If Makerbot Makerware is installed make sure that its background service is not running.
6. Launch ReplicatorG and make sure it properly connects to the printer (top area turns green).
7. Open a STL file in ReplicatorG; when prompted agree to make it touch the build plate.
8. Press Generate Gcode and make the following adjustments as shown in Figure A1.

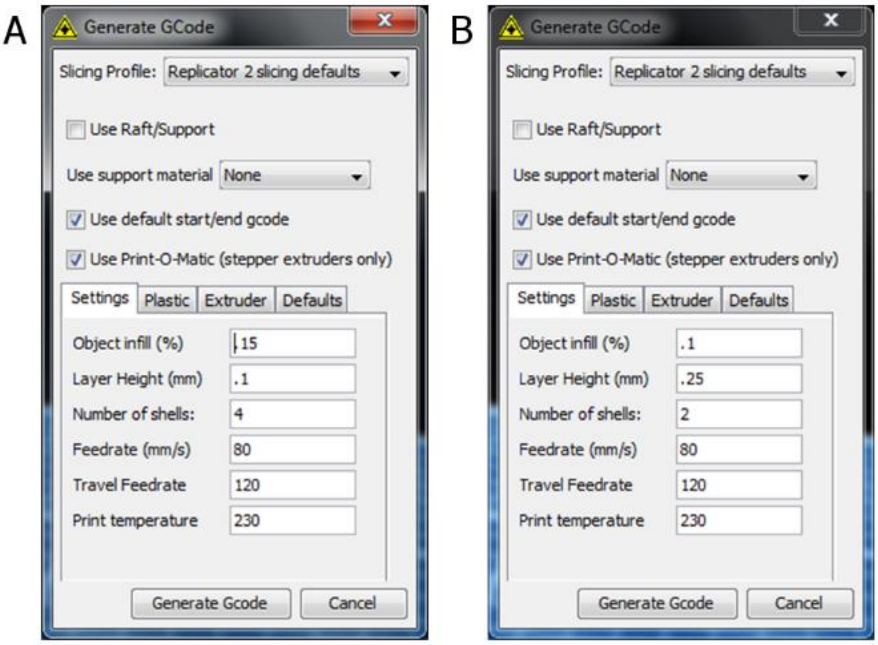

Figure A1: Replicator G settings to generate GCode. (A) Settings for printing pins and (B) for printing the base plate.

9. Press 'Generate Gcode'; the following slicing process may take up to an hour, depending on the PC hardware configuration.
10. When the slicing process is finished start the print by pressing the 'Make' button. Printing will take several hours; while printing occasionally check if the objects properly sticks to the build plate.
11. Carefully remove the finished print from the built plate.
12. Remove excess plastic strings on the finished print using a scalpel (in particular in the holes of the plate and between neighbouring pins).

Orienting embryos for automated imaging

Preparing agarose coated microtiter plates

13. Assemble the orientation tool by sliding the pin stripes into the baseplate.
14. Boil 1% agarose in medium until completely dissolved.
15. Fill 50 µl 1% agarose into each well using a multi-channel pipette.
16. Wait for 1 minute at room temperature.
17. Insert the orientation tool; the clips of the baseplate should be inserted into the corresponding wells.
18. Ensure that all pinstripes are properly inserted and have the same penetration depth.
19. Wait until the agarose has solidified (at least 15 min at room temperature).
20. Carefully remove the orientation tool. Avoid tilting the tool while removing to maintain the position and shape of the agarose plugs.
21. *Optional*: Plates can be stored in a plastic bag at 4°C for a couple of days.

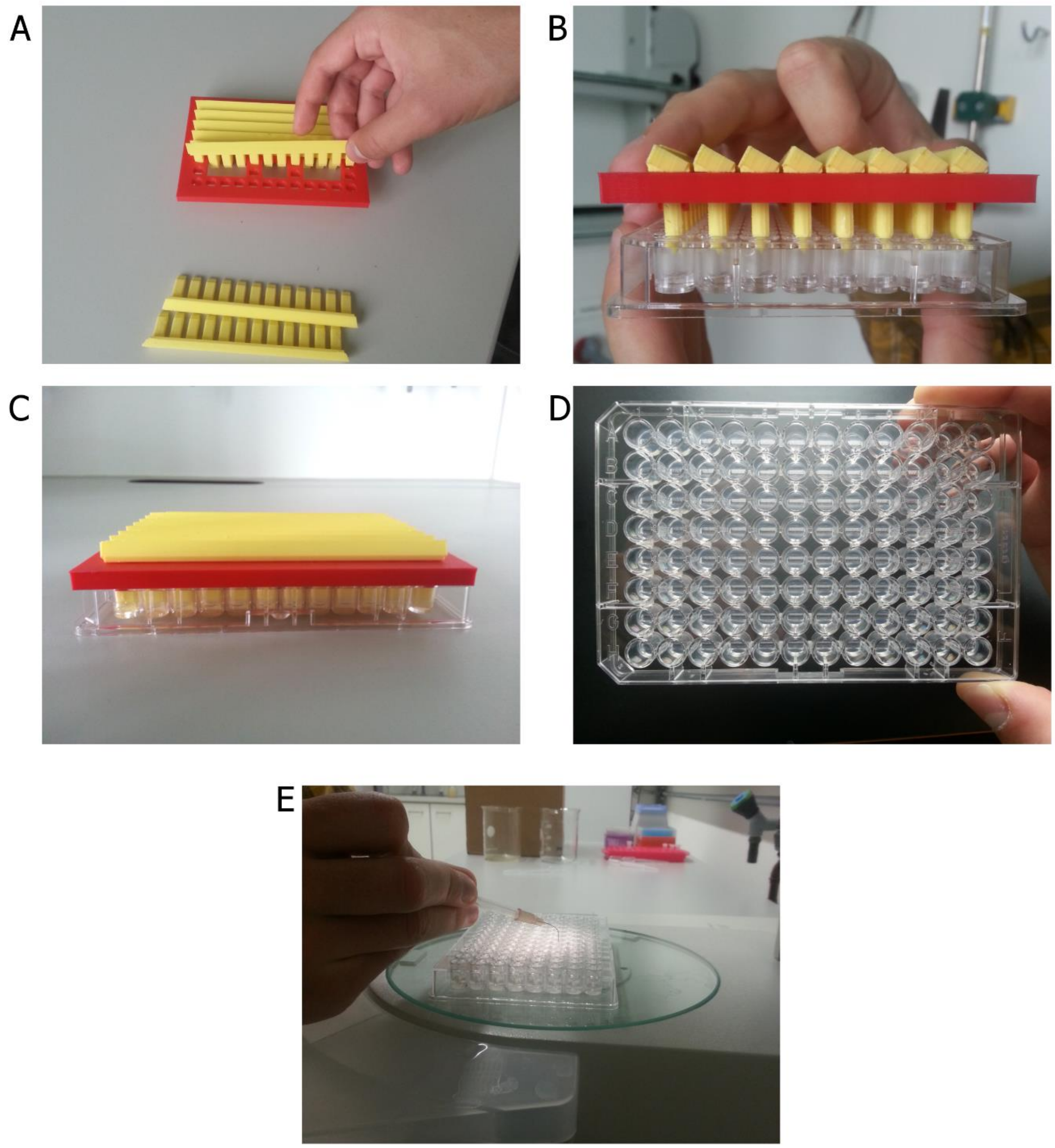

Figure A2: Illustration of generation of agarose coated well plates using 3D printed tools. (A) Assembly of the tool, (B) insertion of tool into microtiter plate containing 50 µl of 1% agarose, (C) inserted tool, (D) agarose-coated plated with cavities for orienting embryos and (E) manual orientation of embryos within cavities.

Preparing embryos for orientation

22. Optional: Treat embryos with 0.003% N-Phenylthiourea starting at 22 hpf.
23. If required dechorionate all embryos using forceps or enzymatically.
24. Anesthetize embryos in 0.003% tricaine in appropriate embryo medium.
25. Transfer embryos in a volume of 100-150µl into wells of agarose coated plates using a cut 200 µl tip or alternative device.

Orientation of embryos

26. Place the embryo containing plate under a stereomicroscope.
27. *Optional*: If possible use oblique illumination to enhance visibility of the agarose cavities.
28. Use a bend injection needle or similar device to position the specimen within the agarose cavities.
29. *Optional:* Orient all embryos with identical anteroposterior positions to have further standardization of datasets.

Dorsal template

30. For automated imaging, position embryos in such a way that all yolk sacs are approximately at the same position within cavities using features of the well plate as guidelines.
31. Adjust tilting of embryos by carefully turning the specimen within the cavity.

Lateral template

32. *Optional:* Orient all embryos in the same dorsoventral orientation to have further standardization of datasets.
33. Insert the yolk ball into the small depression within the cavity.
34. Carefully adjust tilting of specimen.

Requirements

The procedure described here has been optimized for a Makerbot Replicator 2, with a modified extruder (thing:35810) and fan duct (thing:51426). The original build plate was replaced by a 4 mm aluminum plate elevated by a spacer and covered with painters tape. Please refer to the main manuscript for further details.

The protocol has been tested and optimized for usage of the OpenSCAD (http://www.openscad.org/) and ReplicatorG (http://replicat.org/), but should work similarly with any other suitable software packages.
