# Supplementary figures and images for: Generation of orientation tools for automated zebrafish screening assays using desktop 3D printing
Source: BMC Biotechnol. 2014 May 1;14:36. doi: 10.1186/1472-6750-14-36 (PMC4021294; doi:10.1186/1472-6750-14-36)

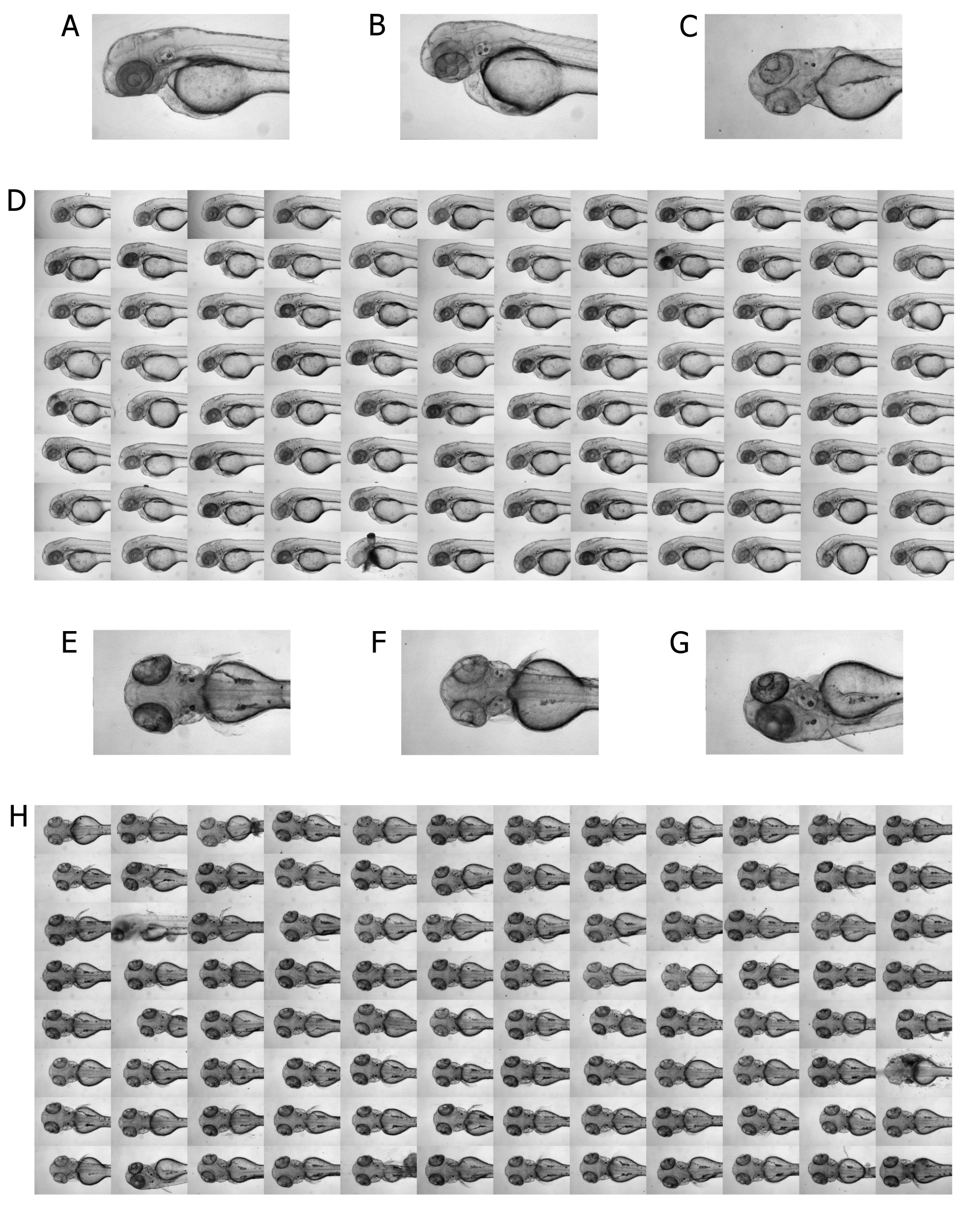

Supplement: Additional file 3: Figure S1 — Examples of data from entire 96 well plates containing oriented embryos. Shown are laterally and dorsally oriented embryos from two single 96 well plates. (A-D) lateral orientation and (E-H) dorsal orientation. To illustrate average orientation accuracy within a single plate originating from a routine experiment, embryos were divided in three categories: (A, E) well oriented (lateral: 92.7%, dorsal: 78.1%), (B, F) slightly tilted (lateral: 6.3%; dorsal: 16.7%) and (C, G) failed orientation (lateral: 1.0%; dorsal: 5.2%). Embryos damaged during pipetting or orienting were included in the failed category. Thumbnail images in D and H show overviews of entire 96 well plates. [file 1472-6750-14-36-S3.tiff]
