# Supplementary material for: Generation of orientation tools for automated zebrafish screening assays using desktop 3D printing
Source: BMC Biotechnol. 2014 May 1;14:36. doi: 10.1186/1472-6750-14-36 (PMC4021294; doi:10.1186/1472-6750-14-36)
Supplement: Additional file 1 — STL files of orientation tool designs. [file 1472-6750-14-36-S1.zip › Additional Material 1/readme.doc]

**Orientation Tool Render and Printing Guide on a Makerbot Replicator 2**

**Using the OpenSCAD scripts**

- Put all three *.scad* files into the same Folder.
- To create the baseplate compile and render (F6) the *baseplate.scad* file, then export as STL (Design -> Export as STL…)
- For the pins open up the *pin.scad* file
  - Adjust the prepare print command according to the syntax given in the file
  - It is recommended to print four stripes at once, more are possible, but may require STL cleaning and/or a powerful machine to render
  - Render the pinstripes and Export as STL

**ReplicatorG**

- Open a STL file in ReplicatorG
- press Generate GCode, in the opening window make the following adjustments:
  - when printing the baseplate

- when printing the pins
